# Anthropogenic pollutant-driven geographical distribution of mesozooplankton communities in estuarine areas of the Bohai Sea, China

**DOI:** 10.1038/s41598-019-46047-5

**Published:** 2019-07-04

**Authors:** Yangchun Gao, Qing Yang, Hongjun Li, Xiaocheng Wang, Aibin Zhan

**Affiliations:** 1 National Marine Environmental Monitoring Center, Dalian, 116023 China; 2 Research Center for Eco-Environmental Sciences, Chinese Academy of Sciences, 18 Shuangqing Road, Haidian District, Beijing, 100085 China; 3 University of Chinese Academy of Sciences, 19A Yuquan Road, Shijingshan District, Beijing, 100049 China

**Keywords:** Animal physiology, Environmental impact

## Abstract

Mesozooplankton communities in marine ecosystems are mainly influenced by both anthropogenic pollutants (e.g. nutrients and heavy metals) and natural variables (e.g. temperature, salinity and geographic distance). To achieve a deeper understanding of the effects of anthropogenic pollutants on mesozooplankton communities, we analyzed the community structure of mesozooplankton from 91 stations representing five typical estuarine regions in the Bohai Sea and assessed the relative importance of anthropogenic pollutants and natural variables by using multiple statistical approaches. Cd was identified as the leading pollutant for observed community variation among the five regions, followed by NH_4_-N and COD. Redundancy analysis (RDA) model demonstrated that mesozooplankton communities were largely determined by both anthropogenic pollutants and natural variables, and the indicator species of mesozooplankton also varied when responding to different factors. Variance partitioning analysis showed both anthropogenic pollutants and natural variables posed significant influences (ANOVA, *P* < 0.05) on the mesozooplankton community structure, but the explanatory power of anthropogenic pollutants overrode the natural variables. These observations highlighted the importance of anthropogenic pollutants in the shifts of zooplankton structures among different regions. Our results obtained in this study provided new insights into the mechanism of the influence of anthropogenic pollutants on mesozooplankton communities in estuarine areas.

## Introduction

The estuarine ecosystems, which link the sea with freshwater habitats, are among the most ecologically and social-economically important globally^1,2^. However, this ecotone have been seriously threatened, particularly in the past half century, by anthropogenic activities, such as massive release of chemical pollutants^3^. The increasing pollution has caused significantly negative effects, such as the losses of marine biodiversity and disturbance of ecological function^4^. Hence, it is crucial to comprehensively study the influence of anthropogenic pollutants on inhabitant organism communities, and the understanding has been considered as great potential to monitor, manage and protect marine biodiversity^5–7^.

The variation of marine communities can be driven by multiple factors such as natural variables and anthropogenic pollutants^8–10^. Mounting evidence demonstrated that natural variables including temperature, salinity and geographic distance play key roles in shaping species richness and abundance^10–14^. Simultaneously, anthropogenic pollutants, such as nutrients and heavy metals, could also influence biodiversity and geographical distribution of marine communities. For example, many anthropogenic pollutants such as ammonia nitrogen and cadmium can pose toxic effects on plankton and fish, thus decreasing the survival rates of sensitive species and reducing species diversity^15–18^. When exploring the influence of anthropogenic pollutants on marine communities, we should also consider the impact of natural variables, which may mask the ecological influence of anthropogenic pollutants^6–10^.

Zooplankton inhabiting coastal ecosystems are key components in the food webs, acting an important trophic link between the marine primary producers and higher trophic levels^3,19^. The crucial role in marine food webs indicates that decline in biodiversity of zooplankton could decrease the survival rates of higher trophic organisms such as fish^20^, and eventually pose far-reaching consequences for the marine food webs^21^. Moreover, zooplankton assemblages consist of species highly sensitive to environmental pollutants such as nutrients and heavy metals, and their variation could be attributed to different levels of pollution in marine systems, such as estuarine systems^19,22,23^ and coastal systems^24,25^, also making them good indicators to explore ecological effects of anthropogenic pollutants. Available studies mainly focused on ecological effects of environmental variables and hydrological processes on zooplankton community structure^26,27^. However, natural variables and anthropogenic pollutants may act synergistically, additively or antagonistically^28–33^, suggesting that it is essential to distinguish respective effects of these two categories of factors.

The Bohai Sea, which is a semi-closed inner sea located in the northeast China, has been impacted by serious pollution caused by anthropogenic activities. The coastal regions support various industries derived from extremely rapid urbanization and industrialization in the past decades, and such rapid development along the coastal regions has caused severe pollution problems to Bohai Sea, especially for the coastal estuaries. The pollution levels varied among the coastal estuaries of Bohai Sea, especially for heavy metal pollutions^3,34–37^. For example, Jinzhou Bay, which is surrounded by one of the old industrial bases in China, was found with the highest concentrations of Cd, the pollution levels gradually decreased along Jinzhou Bay, Luanhekou Estuary, Laizhou Bay, Shuangtaizi Estuary and Bohai Bay^36^. Thus, the environmental gradients along the coastal bays or estuaries provide premise conditions for the exploration of ecological response of zooplankton under anthropogenic stress.

In this study, we aimed to: (1) characterize the community structure of mesozooplankton in the typical Bohai estuarine regions, (2) identify the key factors responsible for local and regional community structure variation, and further (3) explore the respective ecological roles of anthropogenic pollutants and natural variables on community structure.

## Results

### Environmental features

**Figure 1:**
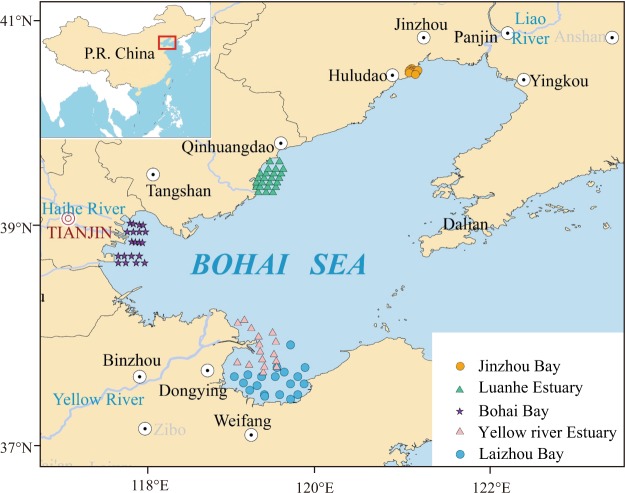
Sampling locations of zooplankton communities along the coastal bays or estuaries of Bohai Sea, China. All maps are made by ArcGIS version 10.0 (ESRI Company, USA).

**Figure 2:**
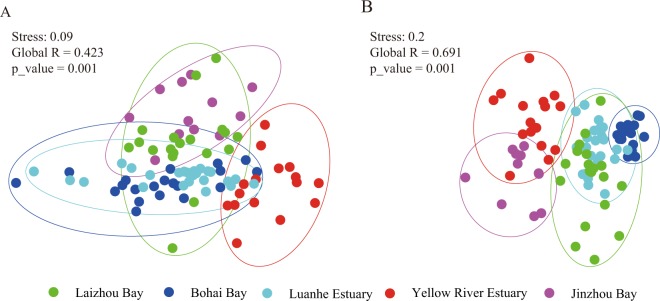
The results of nonmetric multidimensional scaling ordination (NMDS) of environmental variables at each sampling location (**A**) and zooplankton communities at each sampling location (**B**).

Generally, the average value of all environmental factors showed significant difference (*P* < 0.05) among the five regions (Fig. 1; Table 1). Tests based on nonmetric multidimensional scaling (NMDS) and the analysis of similarity (ANOSIM) also provided similar results (Stress = 0.14, Global R = 0.409, *P* = 0.001; Fig. 2A), supporting significant environmental gradients among the five regions.

**Table 1:** Average value and standard deviation and ANOVA test for each environmental factor recorded and sampling dates and coordinate in the five coastal regions of Bohai Sea.

Environmental parameters	Jinzhou Bay	Luanhe Estuary	Bohai Bay	Laizhou Bay	Yellow River Estuary	F	*p* value
Sampling dates	28/08/2015	13/08/2015	4/08/2015	11/08/2015	9/08/2015		
Coordinate	120.9797–121.0789 N 40.7681–40.7800 E	119.2917–119.5900 N 39.4417–39.7833 E	117.7667–118.0667 N 38.6569–39.0967 E	119.0500–119.8300 N 36.7500–37.5000 E	119.0833–119.5167 N 37.4564–38.0333 E		
Temperature (°C)	27.76 ± 0.13^b^	27.08 ± 0.90^b^	28.51 ± 0.0.28^a^	29.16 ± 0.62^a^	27.08 ± 1.07^b^	30.90	<0.000
Salinity	29.52 ± 0.79^b^	30.79 ± 0.46^a^	30.71 ± 0.63^a^	29.33 ± 1.53^b^	28.09 ± 1.79^c^	18.31	<0.001
COD (mg/L)	1.34 ± 0.29^b^	1.36 ± 0.13^b^	2.15 ± 0.51^a^	2.05 ± 0.51^a^	1.57 ± 0.63^b^	14.02	<0.001
Dissolved oxygen (mg/L)	6.32 ± 0.22^b^	7.71 ± 1.05^a^	6.56 ± 1.30^b^	7.04 ± 1.25^ab^	7.33 ± 1.58^ab^	3.91	0.006
Suspended matter (mg/L)	9.87 ± 3.26^b^	20.43 ± 2.05^ab^	22.02 ± 2.83^a^	14.80 ± 8.15^b^	25.74 ± 14.82^a^	10.02	<0.001
PO_4_-P (mg/L)	0.0208 ± 0.0355^a^	0.0076 ± 0.0035^b^	0.0121 ± 0.0047^ab^	0.006 ± 0.005^b^	0.0048 ± 0.004^b^	3.84	0.006
NO_2_-N (mg/L)	0.0404 ± 0.0317^b^	0.0124 ± 0.0051^b^	0.0745 ± 0.05^a^	0.0082 ± 0.0091^c^	0.0615 ± 0.0182^ab^	26.39	<0.001
NO_3_-N (mg/L)	0.2256 ± 0.2633^b^	0.0946 ± 0.0184^b^	0.1897 ± 0.1219^b^	0.5849 ± 0.6757^a^	0.1349 ± 0.0427^b^	7.41	<0.001
NH_4_-N (mg/L)	0.043 ± 0.0318^b^	0.0358 ± 0.0134^b^	0.1226 ± 0.0575^a^	0.0745 ± 0.0616^b^	0.1588 ± 0.0813^a^	17.82	<0.001
Chlorophyll-a (μg/L)	4.1854 ± 1.7692^ab^	5.6525 ± 5.0847^ab^	6.726 ± 5.8175^a^	4.0186 ± 1.8437^ab^	2.6585 ± 5.1404^b^	2.58	0.041
pH	8.15 ± 0.10^a^	8.07 ± 0.09^b^	8.00 ± 0.05^b^	8.08 ± 0.06^ab^	8.01 ± 0.07^b^	12.06	<0.001
As (mg/L)	0.0061 ± 0.0006^a^	0.0012 ± 0.0004^c^	0.0023 ± 0.0003^bc^	0.0026 ± 0.0013^b^	0.0017 ± 0.0006^c^	95.01	<0.001
Hg (mg/L)	0.0001 ± 0^a^	0 ± 0^c^	0 ± 0^c^	0.0001 ± 0^b^	0.0001 ± 0^b^	38.70	<0.001
Cu (mg/L)	0.0034 ± 0.0016^ab^	0.0033 ± 0.0009^ab^	0.0045 ± 0.0026^a^	0.0031 ± 0.0008^b^	0.0016 ± 0.0006^c^	10.72	<0.001
Pb (mg/L)	0.0025 ± 0.0007^a^	0.0002 ± 0.0001^c^	0.0018 ± 0.0017^ab^	0.0011 ± 0.0009^b^	0.0011 ± 0.0005^b^	15.58	<0.001
Cd (mg/L)	0.0007 ± 0.0001^a^	0.0004 ± 0.0001^b^	0.0002 ± 0.0001^c^	0 ± 0.0001^d^	0.0001 ± 0^c^	127.10	<0.001
Zn (mg/L)	0.0243 ± 0.0023^b^	0.0132 ± 0.0029^b^	0.0112 ± 0.0084^b^	0.1724 ± 0.0624^a^	0.0151 ± 0.0046^b^	117.60	<0.001

COD represented chemical oxygen demand.

Different superscript letters indicate significant difference within a row.

Specifically, the concentrations of As, Hg, Pb and Cd were the highest in Jinzhou Bay among the five regions and significantly differed from those in other regions (Table 1). While, the concentration of Zn in Laizhou Bay was significantly higher than that in the rest four regions. The concentration of Cu in Jinzhou Bay, Luanhe Estuary and Bohai Bay was significantly higher than that in Laizhou Bay and Yellow River Estuary. One organic pollutant indicator, COD, in Bohai Bay and Laizhou Bay were significantly higher than that in Yellow River Estuary, Luanhe Estuary and Jinzhou Bay. Luanhe Estuary and Bohai Bay showed significantly higher salinity than that in Jinzhou Bay, Laizhou Estuary and Yellow River Estuary. The concentration of NH_4_-N in Yellow River Estuary and Bohai Bay was significantly higher than that in the rest three regions. While, the concentration of NO_3_-N in Laizhou Bay was highest and significant difference was observed between Laizhou Bay and the other regions (Table 1).

### Zooplankton community structure

**Figure 3:**
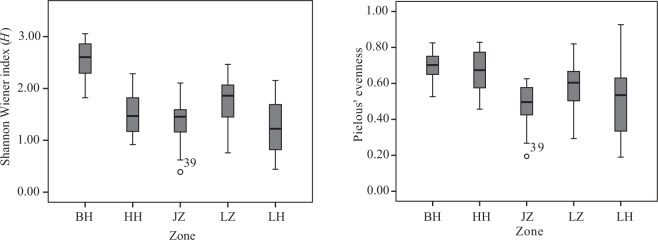
Boxplots of Shannon-Wiener index and Pielou’s evenness of mesozooplankton in the five regions. Boxes, central bars and solid lines indicate the interquartile range, the median and the data range, respectively. The outliers are circles lying outside 1.5 times the interquartile range.

A total of 43 mesozooplankton species were identified in the five regions across all 91 sampling stations (Table 2). The mesozooplankton communities were dominated by *Paracalanus parvus* (26.94%), *Acartia bifilosa* (25.30%), *Oithona similis* (11.50%) and *A. pacifica* (9.21%). When the four diversity index (Species richness, Shannon-Wiener index, Pielous’ evenness and Simpson diversity index) within each region were subjected to the non-parametric Mann-Whitney *U* test, we found significant difference between regions (*P* < 0.05 for most pairs, Table 2). In addition, a coincident result was also observed based on statistical percentiles, as there appeared different median values and 25^th^ and 75^th^ percentiles, such as for Shannon-Wiener index and Pielous’ evenness (Fig. 3).

**Table 2:** Occurrence, density (ind.m^−3^, Mean ± SD) and diversity index of the zooplankton species at each sampling zone in Bohai Sea (Augest 2015).

Species	Bohai Bay	Yellow River Estuary	Jinzhou Bay	Luanhe Estuary	Laizhou Bay	Average
*Paracalanus parvus*	2861.9 ± 2893	30382.8 ± 2077.2	742.4 ± 804.8	2419 ± 1479.3	7049.1 ± 9506.5	3170.5 ± 5061.6
*Acartia bifilosa*	9240.4 ± 12850.4	0 ± 0	0 ± 0	2410 ± 7677.9	1489.3 ± 3985.4	2977.4 ± 8070.4
*Oithona similis*	1283.9 ± 1430.1	13263.4 ± 1093.7	2970.5 ± 1826.4	1703.7 ± 2835.9	404.6 ± 689.5	1353.5 ± 1952.1
*Acartia pacifica*	3397.6 ± 7151.4	20158.7 ± 3781.6	8.4 ± 8	84.3 ± 158.3	444 ± 666.8	1084.3 ± 3870.1
*Oikopleura dioica*	1032.3 ± 1245.7	0 ± 0	0.8 ± 2.9	3098.9 ± 6277.6	0 ± 0	1044.3 ± 3477.6
*Acartia hongi*	0 ± 0	44710.7 ± 3930.4	264.4 ± 284.5	0 ± 0	0 ± 0	526.2 ± 1924.1
*Aidanosagitta crassa*	1468.1 ± 1176.9	2985.9 ± 270.9	150.2 ± 96.3	234.1 ± 159.9	93.1 ± 117.3	456.5 ± 780.4
*Ditrichocorycaeus affinis*	1085.9 ± 1975.2	2788.9 ± 339.7	0 ± 0	85 ± 97.4	65.2 ± 135.6	305.3 ± 1012.4
*Centropages dorsispinatus*	1069.1 ± 1305.9	810 ± 103.3	0 ± 0	8.1 ± 35.7	216.6 ± 441.9	291.2 ± 761.4
*Noctiluca scintillans*	0 ± 0	22723.7 ± 3449	0 ± 0	0 ± 0	18.2 ± 78.9	253.5 ± 1509.1
*Microsetella norvegica*	349.9 ± 739.6	0 ± 0	0 ± 0	119.9 ± 415.3	1.3 ± 5.7	108.8 ± 422.7
*Labidocera euchaeta*	372.4 ± 721.9	256.1 ± 50	2.1 ± 4	0 ± 0	0 ± 0	84.9 ± 366
*Parvocalanus crassirostris*	0 ± 0	0 ± 0	406.9 ± 357.5	0 ± 0	0.8 ± 3.6	53.8 ± 186.5
*Pseudodiaptomus marinus*	38.5 ± 109.2	0 ± 0	0 ± 0	2.2 ± 10.2	1.2 ± 5.4	9.3 ± 52.8
*Eirene ceylonensis*	21.9 ± 22	0 ± 0	2.7 ± 3.9	11 ± 18	0 ± 0	8.1 ± 16.1
*Eirene tenuis*	30.3 ± 23.4	0 ± 0	0 ± 0	0 ± 0	0 ± 0	6.7 ± 16.6
*Labidocera bipinnata*	26.6 ± 70.3	0 ± 0	0 ± 0	0 ± 0	0.1 ± 0.3	5.9 ± 34.2
*Corycaeus japonicus*	0 ± 0	0 ± 0	42.1 ± 104.2	0 ± 0	0 ± 0	5.6 ± 39.1
*Tortanus spinicaudatus*	23.9 ± 40.1	0 ± 0	0 ± 0	0 ± 0	0 ± 0	5.3 ± 20.9
*Pleurobrachia globos*	0 ± 0	0 ± 0	0 ± 0	17.8 ± 37.9	0 ± 0	4.7 ± 20.7
*Obelia dichotoma*	0 ± 0	0 ± 0	0 ± 0	0 ± 0	17.1 ± 55.3	3.6 ± 25.7
*Calanus sinicus*	9.3 ± 19.9	0 ± 0	0 ± 0	0.9 ± 2.5	4.2 ± 12.8	3.1 ± 11.4
*Hydractinia minima*	5 ± 10.3	0 ± 0	0 ± 0	0 ± 0	0 ± 0	1.1 ± 5.2
*Acanthomysis longirostris*	0 ± 0	0 ± 0	5.7 ± 5.5	0 ± 0	0 ± 0	0.8 ± 2.7
*Oithona brevicornis*	0 ± 0	0 ± 0	0 ± 0	2.3 ± 11.1	0 ± 0	0.6 ± 5.7
*Centropages mcmurrichi*	0 ± 0	0 ± 0	0 ± 0	0 ± 0	2.5 ± 11	0.5 ± 5.0
*Evadne tergestina*	0 ± 0	0 ± 0	0 ± 0	0 ± 0	2.5 ± 10.8	0.5 ± 4.9
*Acetes chinensis*	2.3 ± 6.8	0 ± 0	0 ± 0	0 ± 0	0 ± 0	0.5 ± 3.3
*Mysis relicta*	2.2 ± 9.8	0 ± 0	0 ± 0	0 ± 0	0 ± 0	0.5 ± 4.6
*Clytia hemisphaerica*	1.9 ± 5.8	0 ± 0	0 ± 0	0 ± 0	0 ± 0	0.4 ± 2.8
*Penilia avirostris*	0 ± 0	0 ± 0	0 ± 0	1.4 ± 6.6	0 ± 0	0.4 ± 3.4
*Labidocera bipinnata*	0 ± 0	0 ± 0	0 ± 0	1.4 ± 4.2	0 ± 0	0.4 ± 2.2
*Clytia globosa*	1.5 ± 3.6	0 ± 0	0 ± 0	0 ± 0	0 ± 0	0.3 ± 1.8
*Eirene menoni*	1.4 ± 3.1	0 ± 0	0 ± 0	0 ± 0	0 ± 0	0.3 ± 1.5
*Ectopleura dumortieri*	0 ± 0	0 ± 0	0 ± 0	0.5 ± 1.1	0 ± 0	0.1 ± 0.6
*Themisto gracilipes*	0 ± 0	0 ± 0	0.8 ± 2.9	0 ± 0	0 ± 0	0.1 ± 1.0
*Podocoryne minina*	0 ± 0	0 ± 0	0.8 ± 1.6	0 ± 0	0 ± 0	0.1 ± 0.6
*Erythrops minuta*	0.5 ± 2	0 ± 0	0 ± 0	0 ± 0	0 ± 0	0.1 ± 1.0
*Centropages tenuiremis*	0 ± 0	0 ± 0	0 ± 0	0.4 ± 1.4	0 ± 0	0.1 ± 0.7
*Schmackeria poplesia*	0 ± 0	0 ± 0	0 ± 0	0 ± 0	0.4 ± 1.6	0.1 ± 0.7
*Labidocera pavo*	0 ± 0	0 ± 0	0 ± 0	0.3 ± 1.3	0 ± 0	0.1 ± 0.6
*Sarsia nipponica*	0.3 ± 1.1	0 ± 0	0 ± 0	0 ± 0	0 ± 0	0.1 ± 0.5
*Tortanus derjuginii*	0 ± 0	0 ± 0	0.4 ± 0.9	0 ± 0	0 ± 0	0.1 ± 0.3
Species richness	1.28 ± 0.25^a^	0.50 ± 0.16^c^	0.76 ± 0.23^bc^	0.82 ± 0.18^b^	0.64 ± 0.24^c^	
Shannon-Wiener index	2.57 ± 0.32^a^	1.52 ± 0.40^c^	1.35 ± 0.48^c^	1.76 ± 0.43^b^	1.26 ± 0.55^c^	
Peilou’s evenness	0.70 ± 0.08^a^	0.66 ± 0.12^ab^	0.47 ± 0.13^c^	0.59 ± 0.13^b^	0.50 ± 0.21^bc^	
Simpson diversity index	0.77 ± 0.07^a^	0.55 ± 0.13^bc^	0.47 ± 0.17^c^	0.60 ± 0.15^b^	0.44 ± 0.20^c^	

Different superscript letters indicate significant difference (*P* < 0.05) within a column.

The zooplankton communities showed large variation along the environmental gradient based on NMDS and ANOVA analyses (Stress = 0.2, global R = 0.691 with *P* = 0.001; Fig. 2B). Further analyses based on similarity percentage analysis (SIMPER) and pairwise global tests showed zooplankton communities were significantly different among regions (Table S1). The species dissimilarity between regions ranged from 41.23 (global R = 0.662, *P* = 0.001, Bohai Bay versus Luanhe Estuary) to 65.92 (global R = 0.99, *P* = 0.001, Bohai Bay versus Jinzhou Bay), and the average value was 54.95 (Table S1). In addition, the species contribution to the dissimilarity among regions largely varied. For example, the contribution of *P. parvus*, a widely distributed species, ranged from 2.16% (Bohai Bay versus Luanhe Estuary) to 11.43% (Laizhou Bay versus Jinzhou Estuary) and *A. hongi*, another common species, ranged from 4.78% (Bohai Bay versus Jinzhou Bay) to 18.31% (Yellow River Estuary versus Laizhou Bay).

On the other hand, at the intra-regional level, we found a high level of species similarity. The species similarity ranged from 58.26 (Laizhou Bay) to 76.85 (Bohai Bay) with the average value of 65.50 (Table 3). The species contribution to the similarity within each region largely varied, for example, from 6.75% (Jinzhou Bay) to 18.42% (Yellow River Estuary) for *P. parvus* (Table 3).

**Table 3:** Results of the similarity percentage (SIMPER) analysis for each region in Bohai Sea.

	Species	Average Abundance (individual/m^3^)	Average Similarity (%)	Contribution (%)	Cumulative Contribution (%)
Zone Bohai Average similarity:76.85	*Acartia bifilosa*	9240.43	10.53	13.71	13.71
	*Paracalanus parvus*	2861.91	9.24	12.02	25.73
	*Aidanosagitta crassa*	1468.15	8.65	11.25	36.98
	*Acartia pacifica*	3397.64	8.50	11.06	48.05
	*Oithona similis*	1283.91	8.32	10.82	58.87
	*Oikopleura dioica*	1032.29	7.82	10.18	69.05
	*Centropages dorsispinatus*	1069.06	5.61	7.30	76.35
	*Microsetella norvegica*	349.93	4.53	5.89	82.24
	*Ditrichocorycaeus affinis*	1085.89	3.47	4.52	86.76
	*Labidocera euchaeta*	372.38	3.41	4.44	91.19
Zone Yellow River Average similarity:59.64	*Paracalanus parvus*	1898.92	18.42	30.89	30.89
	*Acartia hongi*	2794.42	15.25	25.57	56.45
	*Aidanosagitta crassa*	186.62	9.66	16.19	72.65
	*Oithona similis*	828.96	8.52	14.29	86.94
	*Ditrichocorycaeus affinis*	174.30	3.63	6.08	93.02
Zone Jinzhou Average similarity:64.39	*Oithona similis*	2970.53	23.62	36.68	36.68
	*Aidanosagitta crassa*	150.21	11.73	18.21	54.89
	*Parvocalanus crassirostris*	406.94	8.78	13.64	68.53
	*Paracalanus parvus*	742.43	6.75	10.48	79.02
	*Acartia hongi*	264.40	4.82	7.49	86.50
	*Acartia pacifica*	8.39	3.71	5.76	92.27
Zone Luanhe Average similarity:68.34	*Paracalanus parvus*	2418.99	17.87	26.15	26.15
	*Oithona similis*	1703.75	11.60	16.97	43.12
	*Aidanosagitta crassa*	234.07	11.25	16.47	59.58
	*Acartia bifilosa*	2410.00	11.04	16.16	75.74
	*Oikopleura dioica*	3098.90	7.43	10.87	86.61
	*Ditrichocorycaeus affinis*	85.04	3.12	4.57	91.18
Zone Laizhou Average similarity:58.26	*Acartia bifilosa*	1489.25	13.68	23.48	23.48
	*Paracalanus parvus*	7049.09	13.47	23.13	46.61
	*Aidanosagitta crassa*	93.09	11.16	19.16	65.76
	*Oithona similis*	404.60	8.17	14.02	79.78
	*Acartia pacifica*	444.03	7.00	12.01	91.79

### Explanatory variables for observed community structure

**Figure 4:**
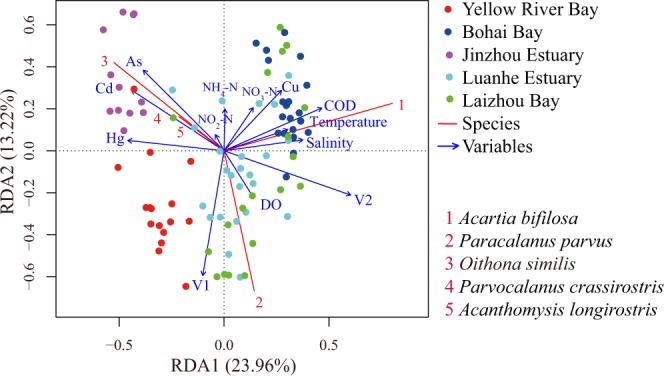
The ordination plot based on redundancy analysis of zooplankton communities. The RDA model (type 2 scaling) explains 49.52% of total variation of mesozooplankton communties with a significant influence (*P* = 0.001). Only the first five top species (1–5 in red color) with the highest contributions to the RDA model are listed.

A total of 17 environmental factors and 18 spatial vectors transformed from geographic coordinate values for sampling stations were considered as possible drivers for structuring observed zooplankton community. Based on forward selection, 13 key factors showed significant influence on the observed community structure (*P* < 0.05, Table S2), including nine anthropogenic pollutants (Cd, NH_4_-N, COD, As, Hg, DO, NO_2_-N, Cu and NO_3_-N) and four natural variables (two spatial variables (V1and V2), salinity and temperature). The selected factors were used for the construction of parsimonious RDA model, which was globally significant (*P* = 0.001) with 49.52% of adjusted R^2^, and the first two axis (RDA1 and RDA2) explained 23.96% and 13.21% of total variations of mesozooplankton communities, respectively (Fig. 4). For anthropogenic pollutants, Cd was the largest contributor in affecting zooplankton community structure, followed by NH_4_-N, COD, As, Hg, DO, NO_2_-N, Cu and NO_3_-N (Fig. 4, Table S2). For example, one high abundance species *O. similis* showed positive correlation with Cd (Fig. 4, Table 2). For natural variables, V2 was the leading contributor of variation in zooplankton community structure, followed by salinity, V1 and temperature (Fig. 4, Table S2). Specifically, *O. similis*, *P. crassirostris* and *A. longirostris* were negatively correlated with V2, whereas *P. parvus* was positively correlated with V1 (Fig. 4). In addition, we detected different influence of anthropogenic pollutants on mesozooplankton. For example, the abundance of *O. similis*, *P. crassirostris* and *Acanthomysis longirostris* were mainly affected by Cd and As, whereas both heavy metals posed no obvious influence on *A. bifilosa* (Fig. 4). Indeed, the abundance of *A. bifilosa* was mainly affected by COD, whereas COD had no obvious influence on *P. parvus* (Fig. 4). Spearson correlation analyses also confirmed this pattern: the abundance of *O. similis* was positively correlated with the concentration of Cd (rho = 0.336, *P* = 0.01, Fig. S1) and *A. bifilosa* was positively correlated with COD (rho = 0.301, *P* = 0.04, Fig. S1).

**Figure 5:**
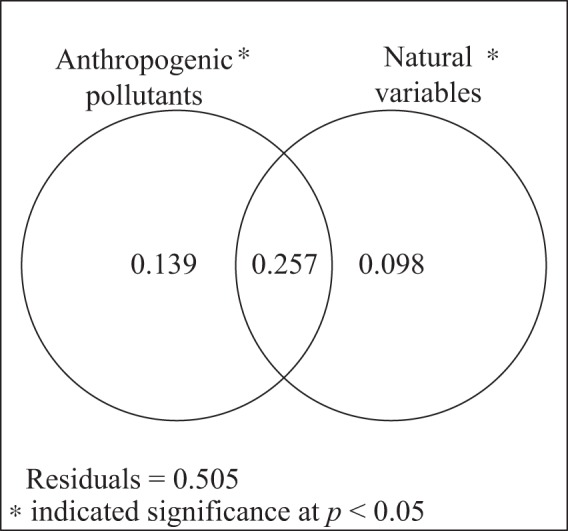
Venn diagram for two explanatory variables. The results of variance partitioning analysis to assess the response of anthropogenic pollutants and natural variables to community structure.

To explore the relative roles of anthropogenic pollutants and natural variables in structuring zooplankton community, variance partitioning was performed for explanatory variables based on nine anthropogenic pollutants (Cd, NH_4_-N, COD, As, Hg, DO, NO_2_-N, Cu and NO_3_-N) and four natural variables (V1, V2, salinity and temperature). The results showed that the shared explained percentage between anthropogenic pollutants and natural variables was 25.7%. Anthropogenic pollutants alone explained 13.9% of the total variation of community structure when excluding the influence of natural variables. Conversely, natural variables alone explained 9.8% of the total variations of community structure when removing the anthropogenic pollutant influence (Fig. 5).

## Discussion

The present study showed that the marine water quality varied largely among the five regions, ranging from very poor for both Jinzhou Bay and Bohai Bay, due to sewage pollution, to good in farming locations such as Luanhe Estuary. Those observations highlighted the differences in their surrounding land-use. A high level of heavy metal pollution was noted in Jinzhou Bay which was characterized with a high level of Cd, As and Hg pollutions in this investigation, mainly due to wastewater discharge by surrounding mining industries in the past century^36^. The organic pollutant concentrations in marine water columns were found to be generally high for Bohai Bay, with high COD being found in the Bohai Bay sampling stations because of sewage discharge from the big cities (Beijing and Tianjin)^38,39^. Whereas the opposite water quality states was observed in Yellow River Estuary, as shown in Fig. 4. Our observations are in agreement with recent studies which have demonstrated that the differences in land-use largely contribute to changes in pollutant variables in aquatic systems^29,36,40^. Hence, the understanding of anthropogenic pollutions induced by different land-use would provide insights into the management of aquatic resource.

Our results clearly showed that both anthropogenic pollutants and natural variables play a crucial role in structuring variation of mesozooplankton communities in the Bohai Sea, the influence of anthropogenic pollutants on mesozooplankton communities overrode that of natural variables. Our RDA model showed that the variations of mesozooplankton communities were in line with the changes in anthropogenic pollutants and natural variables among the five regions, with the effects of anthropogenic pollutants and natural variables being integrated into overall resultant mesozooplankton communities, which was in agreement with recent studies^28,29^. Some species may be sensitive to anthropogenic pollutants, some species may be influenced by natural variables and some other species can be affected by both factors. Those findings were consistent with previous studies in other aquatic systems such as lake, river and marine systems, they have demonstrated that the species‘ responses to environmental factors varied largely^19,22,23,33,41–45^. Thus, different mesozooplankton species responded differently to anthropogenic pollutants and natural variables in the current study, suggesting some species of mesozooplankton communities at different sampling locations can be used as reliable ecological indicators to reflect local environmental pollutions. For example, *O. similis* is a cosmopolitan species, their abundance was positively correlated with the concentration of Cd in the coastal regions of Bohai Sea (*O. similis*-Cd, rho = 0.321, *P* = 0.01). High levels of Cd concentrations were frequently reported in Jinzhou Bay^36,46^, where large numbers of *O. similis* were detected. Whereas, low concentrations of Cd were detected in Bohai Bay and Luanhe Estuary, where few *O. similis* were recorded. Our results indicated that *O. similis* showed high tolerance to Cd pollution and have potential to be a bioindicator of Cd contamination. This founding was in agreement with other studies, they demonstrated that the genera *Oithona* such as *O. nana* could be used as an indicator of anthropogenic pollution^47,48^. *Oithona* was characterized with flexible diet, shorter life cycle and higher reproduction rate compared to other mesozooplankton^48,49^, those physiology features could partly explain its higher tolerance to anthropogenic pollutions.

In addition, *A. bifilosa* showed positive response to COD concentration (*A. bifilosa*-COD, rho = 0.301, *P* = 0.04), indicating the variation of *A. bifilosa* abundance may mirror the fluctuation of COD in marine coastal regions. Indeed, COD contents can reflect the concentrations of anthropogenic organic matters, which can provide aquatic animals with foods and often promote the proliferation and growth of some species of zooplankton communities^50^. Moreover, the abundance of *P. parvus* was negatively correlated with NH_4_-N concentration (Fig. 4), which was also selected by forward selection as an anthropogenic pollutant with significant influences on mesozooplankton communities. High concentration of NH_4_-N can decrease the rates of growth and survival for copepod species^51^. Hence, it is expected that NH_4_-N toxicity can cause similar negative influence on *P. parvus*. However, other factors such as high trophic predators may affect the determinate of bioindicator species, and species may show different tolerant abilities to toxic pollutants under different environments^29^, more studies based on different trophic levels and toxicological validations under laboratory conditions should be performed to verify those findings in the field.

In the current study, Cd was identified as the leading anthropogenic pollutant structuring zooplankton communities in coastal regions of Bohai Sea. Whereas inorganic nitrogen was considered as the main driver for observed zooplankton structure in Tangshan Bay^52^. Moreover, total phosphate, NH_4_-N and Mg^2+^ were identified as the leading factors in Chaobai river, Beiyun river and Fuyang river of China, respectively^41,43,44^. Our results combined with other findings revealed that different leading anthropogenic pollutants were recognized in different ecosystems, even in different regions of the same ecosystems. Those findings indicated that the leading anthropogenic pollutants for observed structure of zooplankton community may be regional-specific, suggesting more works should be performed to clarify driving mechanism of zooplankton structure in regional scale.

Zooplankton community diversity within each region is traditionally used to reflect local environmental conditions^53,54^. Our study showed that Bohai Bay and Jinzhou Bay were both heavily polluted by anthropogenic pollutants. However, two Bays showed large variations in diversity index in the current study. Bohai Bay was mainly suffered from organic pollution, which may pose toxic effects on some species but also may provide more food resources to support more species^55,56^, thus resulted in high species diversity. Jinzhou Bay was mainly polluted by heavy metals, which may only have negative effects on aquatic species, leading to decline of mesozooplankton diversity. Those observations suggested zooplankton community diversity may be not an ideal index to mirror local anthropogenic pollutions, whereas maybe an ideal index to reflect local organic pollutions. Alternatively, the shifts of mesozooplankton community composition in major groups between regions, such as the variation of bioindicator species, may be good indexes to represent local anthropogenic pollutions, as have been revealed at other communities such as benthic communities^29,57,58^.

Traditionally, natural variables including hydrological processes, temperature and salinity are often considered as the major determinants of community structures, and other variables including pollutions played less importance^30–32^. For example, the identification of V2 as leading natural factor indicated that spatial variables such as ocean current may play a crucial role in driving spread of some species, including *O. similis*, *P. crassirostris* and *A. longirostris*, and eventually affect local community structures. However, variance partitioning in our study showed that the explained percentage of community variation by pollutants alone was larger (13.9%) than that by natural variables (9.8%) alone, indicating that anthropogenic pollutants contributed larger than natural variables to the variation of zooplankton community in the coastal regions of Bohai Sea. In the open seas, natural variables such as ocean current and water temperature may play major roles in structuring zooplankton structure^59,60^. Whereas in semi-closed seas such as Bohai Sea, both higher pollution levels^36,46^ and weaker ocean currents^61^ may lead to the observed distribution patterns of mesozooplankton communities in the present study. Thus, the relative roles of anthropogenic pollutants and natural variables in shaping zooplankton structure may largely depend on the relative strengths of both factors. In addition, about quarter of total variations of zooplankton community structure was simultaneously explained by anthropogenic pollutants and natural variables, suggesting strong colinearity between two types of factors exist in the coastal regions of Bohai Sea. Strikingly, *P. parvus* showed positive correlation with V1, dispersal alone cannot explain this observation. One reasonable reason may be that V1 such as ocean current-dispersal largely shaped unmeasured variables or biological processes, which may be actual factor affecting *P. parvus*. Those observations indicated natural variables such as salinity and hydrological processes in this study may affect degradation and dispersal of pollutants^6,46^, highlighting the necessity of excluding the influence of natural variables when exploring the ecological effects of anthropogenic pollutants on zooplankton composition in the field. This idea is especially applicable for marine systems at coastal waters because of complexities of hydrological processes and obvious gradients of natural variables.

In conclusion, our study clearly showed that the mesozooplankton communities among the five regions varied significantly along the environmental gradients. Multiple analyses identified that both anthropogenic pollutants and natural variables were major factors driving mesozooplankton communities in the coastal marine system. Cd was identified the leading anthropogenic pollutants factor structuring mesozooplankton community, followed by Hg, COD, NH_4_-N, As, Zn, NO_2_-N. The species responses to those environmental factors varied largely and mainly depended on organism taxa, suggesting some species can be used as potential bioindicators of environmental pollutants. Further analyses showed that anthropogenic pollutants still played a major role with significant influence on the mesozooplankton community even after removing the natural variable influence, highlighting the necessity of considering negative effects of anthropogenic pollutants on coastal ecosystems in environmental management and monitoring programs. Methodologically, our results emphasized the importance of excluding influence of natural variables including hydrological processes, temperature and salinity when exploring the ecological effects of anthropogenic pollutants on plankton community structure, especially at coastal waters. However, this study was only performed on mesozooplankton that were adequately identified based on morphological features, other zooplankton such as microzooplankton have not been test on this issues. More works on different trophic levels should be carried out using feasible molecular-based methods such as metabarcoding-based identification approach^62^.

## Material and Methods

### Study region and sampling stations

This study focused on five important estuarine areas of the Bohai Sea. The sampling stations mainly distribute in the shallow coastal areas, the water depth of sampling stations is between 2.5 m and 17.0 m. The water column is generally mixed homogeneously due to strong tidal mixing. Neither thermocline nor halocline was observed in the sampling stations of this study because the summertime stratification of the water column mainly occurs in the deep basins (25~35 m depth) in the central Bohai Sea^63^. A total of 91 sample stations were set up over the coastal area of Bohai Sea (Fig. 1), including Jinzhou Bay (12 stations), Luanhe Estuary (24 stations), Bohai Bay (20 stations), Yellow River Estuary (16 stations) and Laizhou Bay (19 stations). Jinzhou Bay, located in northwest of Liaodong Gulf, is a semi-closed shallow water area. Six rivers including Lianshan River, Wuli River, Lao River, Cishan River, Zhouliu River and Tashan River flow into Jinzhou Bay. It is famous as an old industrial base, and become one of the most polluted coastal area in China. Luanhekou Estuary is located on the northwest coast of Bohai Sea with water depths less than 20 m. Freshwater and sediment discharges have decreased greatly since the 1980s due to large dams and reservoirs built along the Luanhe River. Bohai Bay is located on the west of Bohai Sea, near the city of Tianjin and Beijing. Bohai Bay is a typical semi-enclosed coastal area and has limited water exchange with the ocean. Large quantities of industrial and domestic wastewater discharges flow into Bohai Bay from rivers of Beijing-Tianjin. The western coast of Bohai Bay locates the Tianjin Ports, the 10th largest port in the cargo throughout in the world. Yellow River Estuary is located in the southwestern part of Bohai Sea, the end of the second largest river (Yellow River) of the world in terms of sediment load. Yellow River Estuary is characterized with high concentration of Ammonia nitrogen^64^. Laizhou Bay is located on the southern part of Bohai Sea, accounting for up to 10% of the total area. It’s a semi-closed shallow area with average water depth less than 10 m. There are more than a dozen of rivers running into the Laizhou Bay, among which Yellow River, Xiaoqinghe River and Wei River are the most important. All samples were collected in the August, 2015 (Table 1).

### Zooplankton sampling and enumeration

Mesozooplankton samples were quantitatively collected in each sampling station. Specifically, we firstly measured water depth for each sampling station and collected mesozooplankton samples using a plankton net (505 μm mesh size, 50 cm mouth diameter) by towing vertically from 2 m above the bottom to the surface with a speed of 0.5–0.8 m/s. The filtered water volume (m^3^) was measured using the rope length multiplied by the mouth area (0.2 m^2^). The samples were collected and preserved immediately in 5% formaldehyde. In the laboratory, all individuals (zooplankton larvae were not included) were identified into species and enumerated. The abundance (ind./m^3^) of each species was calculated as the number of individuals divided by the filtered water volume. In cases when the mean is presented, the standard deviation was provided (mean ± SD).

### Environmental variable sampling and analysis

Surface seawater samples were collected with a 5 L Niskin bottles from 0.5 m below the surface at each station. The seawater salinity and temperature was measured *in situ* with a multiparameter sensor YSI6600, and pH values were determined with a pH meter. The seawater for dissolved oxygen (DO) analysis was collected with a tube reaching the bottom of bottle until the water overflowed. Suspended matter samples were filtered through pre-weighted Whatman GF/F fiber filters (25 mm). The samples for metal determination were filtered immediately through Whatman GF/F fiber filter (0.45 mm), and then acidified with 10% HNO_3_, placed in an ice box and transported to the laboratory. Concentrations of NO_3_-N, NO_2_-N, NH_4_-N and PO_4_-P in seawater were determined according to the methods described by Grasshoff *et al*.^65^. DO was determined using the Winkler titration method. Chlorophyll-a (Chl-a) was determined by filtering 100–200 mL seawater onto GF/F fiber filter by a cascading filtering device under low vacuum pressure. After extraction with 90% acetone, Chl-a was determined by a Turner Design fluorometer (TD Trilogy). The concentrations of dissolved heavy metals were determined using the inductively coupled plasma mass spectrometry (ICP-MS, Thermo X series) for Cd, Pb, Zn and Cu, while the content of Hg and As was determined using the atomic fluorescence spectrometer (AFS-920).

### Spatial variables

Besides environmental variables, the variations on the spatial distribution of aquatic communities are traditionally correlated with geographical distances between sampling stations^43,44,66^. The spatial distances were generated based on Cartesian coordinates and Euclidian distance matrix, which were transformed from longitude and latitude among the sampling stations. In detail, the longitude and latitude were converted to Cartesian coordinates using the geoXY function available in the SoDa packages in R software v.3.4.1^67^. Then, an Euclidian distance matrix on this Cartesian coordinates was computed using the *dist* function and *PCNM* (Principal Coordinates of Neighbor Matrices) analysis (permutations = 1000) was performed on this matrix using *PCNM* function implemented in the *PCNM* package. The method of PCNM^68,69^ can effectively model spatial structure in biological communities among sampling stations^70^ and has been increasingly used in various groups including bacteria and phytoplankton^71,72^. In this study, we attempted to apply the method of PCNM to mesozooplankton in order to understand the effects of spatial variables on mesozooplankton community. The number of PCNM variables formed is always dependent on the number of sampling stations and their spatial relations. At last, a total of 18 PCNM vectors (V1-V18) showing positive spatial autocorrelation were formed and used as spatial variables for subsequent redundancy analysis (RDA) and forward selection. In detail, the first PCNM vectors indicate spatial relations among sampling stations at a large scale (e.g. between sampling stations across regions) and the last PCNM vectors represent spatial relations among a small scale (e.g. between sampling stations in the same region).

### Statistics analysis

In order to separate the effects of anthropogenic pollutants on mesozooplankton communities from natural environmental factors, the 17 environmental variables together with 18 spatial variables were reclassified into two groups: natural variables (temperature, salinity, and spatial variables) and anthropogenic pollutants (COD, suspend matters, DO, Chl-a, pH, PO_4_, NO_2_-N, NO_3_-N, NH_4_-N, As, Hg, Cu, Pb, Cd, and Zn). The average value and standard deviation for each environmental variable and study location were calculated. One-way ANOVAs were used to compare means of environmental variables among study locations, after testing for homogeneity of variances (Levene’s test, *P* < 0.05) and normality of distribution (Shapiro-Wilk test, *P* < 0.05) using Paleontological Statistics (PAST) version 3.01^73^. Significant ANOVAs (*P* < 0.05) were followed by Tukey HSD post hoc analysis to identify differences between study locations using PAST version 3.01.

Before statistical analyses, all measured environmental factors (except for pH) and mesozooplankton data were log10 (x + 1) transformed to improve normality. To characterize distribution patterns of zooplankton, the composition and abundance of zooplankton were analyzed using non-parametric multivariate methods implemented in PRIMER 5.0^74^. The abundance of zooplankton between regions was compared using nonmetric multidimensional scaling (NMDS) and the analysis of similarity (ANOSIM), which is based on Bray-Curtis distance and rank dissimilarity. The major species driving distribution patterns of zooplankton assemblages at both inter regions and intra regions were identified using similarity percentage analysis (SIMPER) with a cutoff of 90% contributions. The NMDS, ANOSIM and SIMPER analyses were performed using PRIMER 5.0^74^

To recognize the major factors responsible for observed zooplankton community structure, we performed the linear ordination method of RDA, which was chosen mainly based on a preliminary detrended correspondence analysis (DCA) on zooplankton community. The DCA showed that the longest length of gradient (3.03) was shorter than four, indicating that the majority of taxa showed a linear response to explanatory variables^75^. To avoid multicollinearity problems and construct parsimonious RDA model, which has been proved to have greater predictive power for the relationship between zooplankton communities and explanatory variables^76^, we conducted forward selection to select significant explanatory variables including environmental factors and spatial variables using the *forward.sel* function (ANOVAS; 1000 permutations) in *packfor* package in R, which simultaneously taken account for significance (*P* < 0.05) and adjusted R^2^ of the global RDA model with all available explanatory variables^77^. To verify the correlations obtained from RDA analysis, additional Spearman correlation analysis was also performed.

To evaluate the ecological effects of anthropogenic pollutons on mesozooplankton structures, variance partitioning and partial redundancy analysis (pRDA) were performed to estimate explained percentage of the significant anthropogenic pollutions and natural variables selected by forward selection. Variance analyses (ANOVAS; 1000 permutations) were performed to test the significance of RDA and pRDA. Those analyses including RDA, pRDA, ANOVA and DCA analyses were computed using *vegan* package in R software.

## Supplementary information

Supplementary information
