# Supplementary material for: Anthropogenic pollutant-driven geographical distribution of mesozooplankton communities in estuarine areas of the Bohai Sea, China
Source: Sci Rep. 2019 Jul 4;9:9668. doi: 10.1038/s41598-019-46047-5 (PMC6609641; doi:10.1038/s41598-019-46047-5)
Supplement: Supplementary file 1 — Supplementary information [file 41598_2019_46047_MOESM1_ESM.docx]

**Anthropogenic pollutant-driven geographical distribution of mesozooplankton communities in estuarine areas of the Bohai Sea, China**

Yangchun Gao^1,2,3^, Qing Yang^1^, Hongjun Li^1^, Xiaocheng Wang^1^, Aibin Zhan^2,3^

^1^ National Marine Environmental Monitoring Center, Dalian 116023, China.

^2^ Research Center for Eco-Environmental Sciences, Chinese Academy of Sciences, 18 Shuangqing Road, Haidian District, Beijing 100085, China.

^3^ University of Chinese Academy of Sciences, 19A Yuquan Road, Shijingshan District, Beijing 100049, China.

Correspondence and requests for materials should be addressed to H.L. (email: [hjli@nmemc.org.cn](mailto:hjli@nmemc.org.cn))


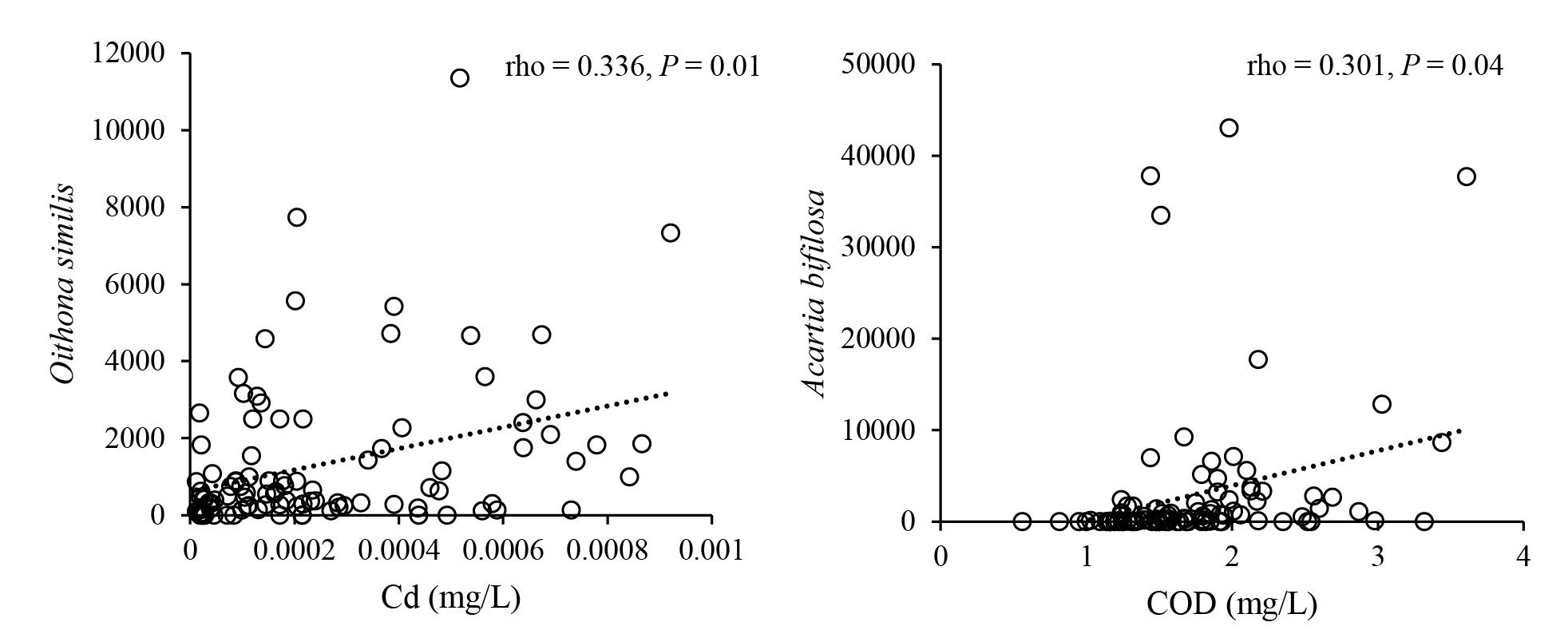
**Fig. S1** Spearson correlation analysis between zooplankton and environmental factors (*O. similis*-Cd, *A. bifilosa*-COD)

**Table S1** Results of the similarity percentage (SIMPER) analysis among the five regions and the analysis of similarity (ANOSIM) of variance between the five zones in the Bohai Sea.

| Bohai Bay versus Yellow River Estuary (Global test, R=0.93, *P* value=0.001)  Average dissimilarity=62.29 | | | | |
| --- | --- | --- | --- | --- |
| Species | Average abundance | | Contribution % | Cumulative contribution % |
|  | Bohai Bay | Yellow River Estuary |  |  |
| *Acartia bifilosa* | 9240.43 | 0.00 | 13.04 | 13.04 |
| *Oikopleura dioica* | 1032.29 | 0.00 | 9.86 | 22.90 |
| *Acartia hongi* | 0.00 | 2794.42 | 9.50 | 32.40 |
| *Acartia pacifica* | 3397.64 | 1259.92 | 8.49 | 40.89 |
| *Microsetella norvegica* | 349.93 | 0.00 | 6.98 | 47.87 |
| *Centropages dorsispinatus* | 1069.06 | 50.63 | 6.73 | 54.60 |
| *Ditrichocorycaeus affinis* | 1085.89 | 174.30 | 5.56 | 60.16 |
| *Labidocera euchaeta* | 372.38 | 16.00 | 5.49 | 65.65 |
| *Aidanosagitta crassa* | 1468.15 | 186.62 | 4.71 | 70.36 |
| *Oithona similis* | 1283.91 | 828.96 | 4.65 | 75.01 |
| *Eirene tenuis* | 30.34 | 0.00 | 4.61 | 79.63 |
| *Eirene ceylonensis* | 21.91 | 0.00 | 3.28 | 82.90 |
| *Paracalanus parvus* | 2861.91 | 1898.92 | 2.74 | 85.65 |
| *Noctiluca scintillans* | 0.00 | 1420.23 | 2.73 | 88.38 |
| *Pseudodiaptomus marinus* | 38.48 | 0.00 | 2.24 | 90.62 |
| Bohai Bay versus Jinzhou Bay (Global test, R=0.99, *P* value=0.001)  Average dissimilarity=65.92 | | | | |
| Species | Average abundance | | Contribution % | Cumulative contribution % |
|  | Bohai Bay | Jinzhou Bay |  |  |
| *Acartia bifilosa* | 9240.43 | 0.00 | 12.05 | 12.05 |
| *Oikopleura dioica* | 1032.29 | 0.83 | 8.77 | 20.82 |
| *Centropages dorsispinatus* | 1069.06 | 0.00 | 7.83 | 28.65 |
| *Acartia pacifica* | 3397.64 | 8.39 | 7.57 | 36.22 |
| *Microsetella norvegica* | 349.93 | 0.00 | 6.45 | 42.67 |
| *Parvocalanus crassirostris* | 0.00 | 406.94 | 6.37 | 49.03 |
| *Ditrichocorycaeus affinis* | 1085.89 | 0.00 | 6.25 | 55.29 |
| *Labidocera euchaeta* | 372.38 | 2.08 | 5.13 | 60.41 |
| *Paracalanus parvus* | 2861.91 | 742.43 | 5.09 | 65.50 |
| *Acartia hongi* | 0.00 | 264.40 | 4.78 | 70.28 |
| *Eirene tenuis* | 30.34 | 0.00 | 4.26 | 74.54 |
| *Aidanosagitta crassa* | 1468.15 | 150.21 | 3.49 | 78.03 |
| *Eirene ceylonensis* | 21.91 | 2.65 | 2.75 | 80.79 |
| *Pseudodiaptomus marinus* | 38.48 | 0.00 | 2.07 | 85.23 |
| *Tortanus spinicaudatus* | 23.90 | 0.00 | 2.03 | 87.26 |
| *Acanthomysis longirostris* | 0.00 | 5.72 | 1.98 | 89.24 |
| *Oithona similis* | 1283.91 | 2970.53 | 1.91 | 91.15 |
| Yellow River Estuary versus Jinzhou Bay (Global test, R=0.50, *P* value=0.001)  Average dissimilarity=53.29 | | | | |
| Species | Average abundance | | Contribution % | Cumulative contribution % |
|  | Yellow River Estuary | Jinzhou Bay |  |  |
| *Parvocalanus crassirostris* | 0.00 | 406.94 | 13.36 | 13.36 |
| *Acartia hongi* | 2794.42 | 264.40 | 12.31 | 25.67 |
| *Oithona similis* | 828.96 | 2970.53 | 11.19 | 36.87 |
| *Paracalanus parvus* | 1898.92 | 742.43 | 11.11 | 47.97 |
| *Acartia pacifica* | 1259.92 | 8.39 | 8.55 | 56.52 |
| *Ditrichocorycaeus affinis* | 174.30 | 0.00 | 7.83 | 64.35 |
| *Aidanosagitta crassa* | 186.62 | 150.21 | 5.88 | 70.24 |
| *Centropages dorsispinatus* | 50.63 | 0.00 | 5.11 | 75.35 |
| *Noctiluca scintillans* | 1420.23 | 0.00 | 4.92 | 85.24 |
| *Acanthomysis longirostris* | 0.00 | 5.72 | 4.14 | 89.38 |
| *Labidocera euchaeta* | 16.00 | 2.08 | 2.93 | 92.31 |
| Bohai Bay versus Luanhe Estuary (Global test, R=0.66, *P* value=0.001)  Average dissimilarity=41.23 | | | | |
| Species | Average abundance | | Contribution % | Cumulative contribution % |
|  | Bohai Bay | Luanhe Estuary |  |  |
| *Centropages dorsispinatus* | 1069.06 | 8.07 | 11.12 | 11.12 |
| *Acartia pacifica* | 3397.64 | 84.28 | 9.96 | 21.08 |
| *Labidocera euchaeta* | 372.38 | 0.00 | 8.24 | 29.32 |
| *Microsetella norvegica* | 349.93 | 119.94 | 8.21 | 37.54 |
| *Ditrichocorycaeus affinis* | 1085.89 | 85.04 | 7.52 | 45.06 |
| *Oikopleura dioica* | 1032.29 | 3098.90 | 6.38 | 51.44 |
| *Acartia bifilosa* | 9240.43 | 2410.00 | 6.34 | 57.78 |
| *Eirene tenuis* | 30.34 | 0.00 | 6.33 | 64.11 |
| *Aidanosagitta crassa* | 1468.15 | 234.07 | 4.03 | 68.15 |
| *Eirene ceylonensis* | 21.91 | 10.98 | 4.03 | 72.17 |
| *Oithona similis* | 1283.91 | 1703.75 | 3.90 | 76.07 |
| *Pseudodiaptomus marinus* | 38.48 | 2.21 | 3.20 | 79.27 |
| *Tortanus spinicaudatus* | 23.90 | 0.00 | 3.03 | 82.30 |
| *Pleurobrachia globos* | 0.00 | 17.77 | 2.82 | 85.12 |
| *Labidocera bipinnata* | 26.61 | 0.00 | 2.30 | 87.42 |
| *Paracalanus parvus* | 2861.91 | 2418.99 | 2.16 | 89.58 |
| *Calanus sinicus* | 9.27 | 0.89 | 2.16 | 91.74 |
| Yellow River Estuary versus Luanhe Estuary (Global test, R=0.80, *P* value=0.001)  Average dissimilarity=55.87 | | | | |
| Species | Average abundance | | Contribution % | Cumulative contribution % |
|  | Yellow River Estuary | Luanhe Estuary |  |  |
| *Acartia hongi* | 2794.42 | 0.00 | 15.70 | 15.70 |
| *Acartia bifilosa* | 0.00 | 2410.00 | 14.58 | 30.27 |
| *Oikopleura dioica* | 0.00 | 3098.90 | 12.69 | 42.96 |
| *Oithona similis* | 828.96 | 1703.75 | 8.24 | 51.20 |
| *Acartia pacifica* | 1259.92 | 84.28 | 7.95 | 59.16 |
| *Ditrichocorycaeus affinis* | 174.30 | 85.04 | 7.12 | 66.28 |
| *Aidanosagitta crassa* | 186.62 | 234.07 | 4.88 | 71.16 |
| *Centropages dorsispinatus* | 50.63 | 8.07 | 4.57 | 75.73 |
| *Paracalanus parvus* | 1898.92 | 2418.99 | 4.32 | 80.05 |
| *Noctiluca scintillans* | 1420.23 | 0.00 | 4.24 | 84.29 |
| *Eirene ceylonensis* | 0.00 | 10.98 | 3.36 | 87.65 |
| *Pleurobrachia globos* | 0.00 | 17.77 | 3.35 | 91.01 |
| Jinzhou Bay versus Luanhe Estuary (Global test, R=0.90, *P* value=0.001)  Average dissimilarity=57.06 | | | | |
| Species | Average abundance | | Contribution % | Cumulative contribution % |
|  | Jinzhou Bay | Luanhe Estuary |  |  |
| *Acartia bifilosa* | 0.00 | 2410.00 | 13.80 | 13.80 |
| *Oikopleura dioica* | 0.83 | 3098.90 | 11.80 | 25.60 |
| *Parvocalanus crassirostris* | 406.94 | 0.00 | 10.68 | 36.28 |
| *Paracalanus parvus* | 742.43 | 2418.99 | 9.00 | 45.28 |
| *Acartia hongi* | 264.40 | 0.00 | 7.96 | 53.24 |
| *Ditrichocorycaeus affinis* | 0.00 | 85.04 | 6.38 | 59.62 |
| *Oithona similis* | 2970.53 | 1703.75 | 5.66 | 65.28 |
| *Acartia pacifica* | 8.39 | 84.28 | 5.07 | 70.35 |
| *Eirene ceylonensis* | 2.65 | 10.98 | 3.34 | 77.68 |
| *Acanthomysis longirostris* | 5.72 | 0.00 | 3.31 | 80.99 |
| *Aidanosagitta crassa* | 150.21 | 234.07 | 3.25 | 84.24 |
| *Pleurobrachia globos* | 0.00 | 17.77 | 3.18 | 87.42 |
| *Microsetella norvegica* | 0.00 | 119.94 | 2.90 | 90.32 |
| Bohai Bay versus Laizhou Bay (Global test, R=0.68, *P* value=0.001)  Average dissimilarity=50.68 | | | | |
| Species | Average abundance | | Contribution % | Cumulative contribution % |
|  | Bohai Bay | Laizhou Bay |  |  |
| *Oikopleura dioica* | 1032.29 | 0.00 | 12.11 | 12.11 |
| *Microsetella norvegica* | 349.93 | 1.32 | 8.32 | 20.43 |
| *Centropages dorsispinatus* | 1069.06 | 216.63 | 7.51 | 27.95 |
| *Labidocera euchaeta* | 372.38 | 0.00 | 7.36 | 35.31 |
| *Ditrichocorycaeus affinis* | 1085.89 | 65.15 | 7.26 | 42.56 |
| *Acartia pacifica* | 3397.64 | 444.03 | 6.94 | 49.50 |
| *Acartia bifilosa* | 9240.43 | 1489.25 | 6.12 | 55.62 |
| *Aidanosagitta crassa* | 1468.15 | 93.09 | 5.74 | 61.36 |
| *Paracalanus parvus* | 2861.91 | 7049.09 | 5.73 | 67.09 |
| *Eirene tenuis* | 30.34 | 0.00 | 5.67 | 72.76 |
| *Oithona similis* | 1283.91 | 404.60 | 5.66 | 78.42 |
| *Eirene ceylonensis* | 21.91 | 0.00 | 4.03 | 82.45 |
| *Pseudodiaptomus marinus* | 38.48 | 1.23 | 2.84 | 85.29 |
| *Tortanus spinicaudatus* | 23.90 | 0.00 | 2.69 | 87.98 |
| *Labidocera bipinnata* | 26.61 | 0.07 | 2.08 | 90.06 |
| Yellow River Estuary versus Laizhou Bay (Global test, R=0.54, *P* value=0.001)  Average dissimilarity=56.75 | | | | |
| Species | Average abundance | | Contribution % | Cumulative contribution % |
|  | Yellow River Estuary | Laizhou Bay |  |  |
| *Acartia hongi* | 2794.42 | 0.00 | 18.31 | 18.31 |
| *Acartia bifilosa* | 0.00 | 1489.25 | 16.15 | 34.46 |
| *Acartia pacifica* | 1259.92 | 444.03 | 11.23 | 45.69 |
| *Paracalanus parvus* | 1898.92 | 7049.09 | 10.42 | 56.11 |
| *Oithona similis* | 828.96 | 404.60 | 9.99 | 66.10 |
| *Centropages dorsispinatus* | 50.63 | 216.63 | 7.99 | 74.10 |
| *Ditrichocorycaeus affinis* | 174.30 | 65.15 | 7.98 | 82.07 |
| *Noctiluca scintillans* | 1420.23 | 18.18 | 5.37 | 87.45 |
| *Aidanosagitta crassa* | 186.62 | 93.09 | 5.23 | 92.68 |
| Jinzhou Bay versus Laizhou Bay (Global test, R=0.73, *P* value=0.001)  Average dissimilarity=61.98 | | | | |
| Species | Average abundance | | Contribution % | Cumulative contribution % |
|  | Jinzhou Bay | Laizhou Bay |  |  |
| *Acartia bifilosa* | 0.00 | 1489.25 | 14.21 | 14.21 |
| *Paracalanus parvus* | 742.43 | 7049.09 | 11.43 | 25.63 |
| *Parvocalanus crassirostris* | 406.94 | 0.82 | 11.36 | 36.99 |
| *Oithona similis* | 2970.53 | 404.60 | 10.53 | 47.52 |
| *Acartia hongi* | 264.40 | 0.00 | 8.52 | 56.04 |
| *Acartia pacifica* | 8.39 | 444.03 | 7.95 | 63.99 |
| *Centropages dorsispinatus* | 0.00 | 216.63 | 6.44 | 70.44 |
| *Ditrichocorycaeus affinis* | 0.00 | 65.15 | 4.38 | 74.82 |
| *Aidanosagitta crassa* | 150.21 | 93.09 | 4.19 | 83.29 |
| *Acanthomysis longirostris* | 5.72 | 0.00 | 3.56 | 86.85 |
| *Eirene ceylonensis* | 2.65 | 0.00 | 2.01 | 88.86 |
| *Corycaeus japonicus* | 42.11 | 0.00 | 1.88 | 90.74 |
| Luanhe Estuary versus Laizhou Bay (Global test, R=0.38, *P* value=0.001)  Average dissimilarity=44.43 | | | | |
| Species | Average abundance | | Contribution % | Cumulative contribution % |
|  | Luanhe Estuary | Laizhou Bay |  |  |
| *Oikopleura dioica* | 3098.90 | 0.00 | 15.95 | 15.95 |
| *Acartia pacifica* | 84.28 | 444.03 | 10.05 | 26.01 |
| *Oithona similis* | 1703.75 | 404.60 | 10.02 | 36.02 |
| *Paracalanus parvus* | 2418.99 | 7049.09 | 9.92 | 45.94 |
| *Ditrichocorycaeus affinis* | 85.04 | 65.15 | 8.61 | 54.55 |
| *Centropages dorsispinatus* | 8.07 | 216.63 | 8.11 | 62.66 |
| *Acartia bifilosa* | 2410.00 | 1489.25 | 8.04 | 70.71 |
| *Aidanosagitta crassa* | 234.07 | 93.09 | 5.17 | 75.87 |
| *Eirene ceylonensis* | 10.98 | 0.00 | 4.22 | 80.10 |
| *Pleurobrachia globos* | 17.77 | 0.00 | 4.22 | 84.32 |
| *Microsetella norvegica* | 119.94 | 1.32 | 4.13 | 88.45 |
| *Calanus sinicus* | 0.89 | 4.18 | 1.82 | 90.27 |
| The signficant tests between zones were performed using one-way ANOVA test. | | | |  |

**Table S2** Anthropogenic pollutants and natural variables selected by forward selection

|  | variables | R^2^ | R^2^Cum | AdjR^2^Cum | F | *P*val |
| --- | --- | --- | --- | --- | --- | --- |
| Anthropogenic pollutants | Cd | 0.11899553 | 0.1189955 | 0.1090966 | 12.021054 | 0.00009999 |
|  | NH4-N | 0.08540784 | 0.2044034 | 0.1863216 | 9.44686 | 0.00009999 |
|  | COD | 0.06647551 | 0.2708789 | 0.2457368 | 7.931974 | 0.00009999 |
|  | As | 0.06652655 | 0.3374054 | 0.3065871 | 8.634666 | 0.00009999 |
|  | Hg | 0.03344179 | 0.3708472 | 0.3338382 | 4.518064 | 0.00089991 |
|  | Oxygen | 0.02596318 | 0.3968104 | 0.3537254 | 3.615625 | 0.00329967 |
|  | NO2-N | 0.02554865 | 0.4223591 | 0.3736424 | 3.671031 | 0.00209979 |
|  | Cu | 0.01730834 | 0.4396674 | 0.3850008 | 2.532932 | 0.02259774 |
|  | NO3-N | 0.01747242 | 0.4571398 | 0.396822 | 2.607055 | 0.02429757 |
| Natural variables | V2 | 0.16491467 | 0.1649147 | 0.1555317 | 17.57593 | 1.00E-04 |
|  | Salinity | 0.09070592 | 0.2556206 | 0.2387029 | 10.72319 | 1.00E-04 |
|  | V1 | 0.0838634 | 0.339484 | 0.3167076 | 11.04608 | 1.00E-04 |
|  | Temperature | 0.04488248 | 0.3843665 | 0.3557323 | 6.26979 | 1.00E-04 |
